# Editorial: Cognitive and Brain Aging: Interventions to Promote Well-Being in Old Age

**DOI:** 10.3389/fnagi.2019.00268

**Published:** 2019-10-15

**Authors:** Philip P. Foster, Carryl L. Baldwin, James Campbell Thompson, Thomas Espeseth, Xiong Jiang, Pamela M. Greenwood

**Affiliations:** ^1^Pulmonary Section, Department of Medicine, Center for Space Medicine, Baylor College of Medicine, Houston, TX, United States; ^2^Department of Chemistry, Rice University, Houston, TX, United States; ^3^Department of Medicine, McGovern Medical School, University of Texas, Houston, TX, United States; ^4^Department of Mathematics and Statistics, University of Houston–Clear Lake, Houston, TX, United States; ^5^George Mason University, Fairfax, VA, United States; ^6^University of Oslo, Oslo, Norway; ^7^Georgetown University, Washington, DC, United States

**Keywords:** mindfulness, meditation, tDCS, neurofeedback, smartphone apps software, exergaming

## Roadmap for Interventions Preventing Cognitive Aging

### The Research Topic in Brief

Collectively, the studies of this book and the associated data sets provide an unprecedented resource for understanding the aging of the brain, the brain networks plasticity, and structure. A particular focus of this book is about the hippocampal and neocortical circuits with the emphasis on protective and enhancement factors of cognition. Major protective interventions evaluated are memory training programs, executive semantic processing of words, foreign-language learning, behavioral cognitive training, attention, mindfulness and transcendental meditation, transcranial direct current stimulation, neurofeedback, physical activity-aerobic exercise, dancing, and diet. There is also an analysis of the relation obesity-cognition and interventions of interest. The state-of-the-art training devices and software currently in clinical trials waiting for FDA approval are outlined. The relativity of the internal perception of time accelerating in aging brains is examined.

### Cognition Defined

✓ ***Cognition*** is the study of mental processes of acquiring knowledge and understanding, enabling critical thinking, problem solving, language, creativity, social intelligence, and mastering fundamental concepts like the mere idea of number, time, quantity, causality, and justice (Piaget and Inhelder, 1947; Chomsky, 1964; Hauser et al., 2002; Piaget, 2009; Mattson, 2019).
✓ ***Psychological testing***(psychometrics) evaluates human abilities, attitudes, traits, and learning evolution based on standards of objectivity, reliability, and validity (Piaget and Inhelder, 1947; Piaget, 2009). In this book, the authors are frequently studying and testing various aspects of memory processing and storage, such as episodic, working memory, and spatial memory. Logical thinking is also assessed via elaborated tasks: the term “fluid intelligence” is also referred to by the authors for logical problem solving (Greenwood and Parasuraman, 2016).
✓ ***Neuropsychological tests***are specifically designed to determine the particular brain structure or pathway activated, e.g., cerebral imaging.

### Brain Mapping and Network Neuroscience

**Figure 1:**
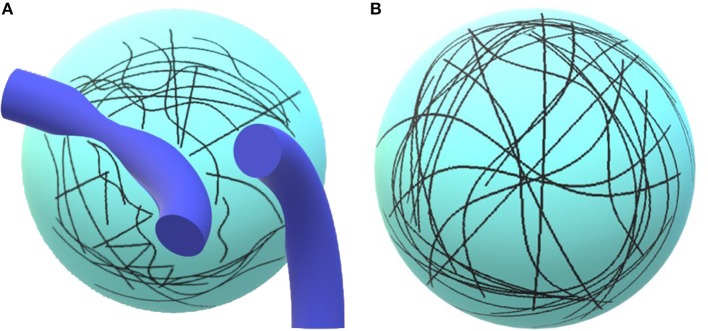
Illustration of the concept. On **(A)**, the diffusion of water molecules is limited by tissues, e.g., vessels, axons, or dendrites. The trajectories are short. **(B)** Paucity of tissues, water molecules may freely diffuse and follow long trajectories.

**Figure 2:**
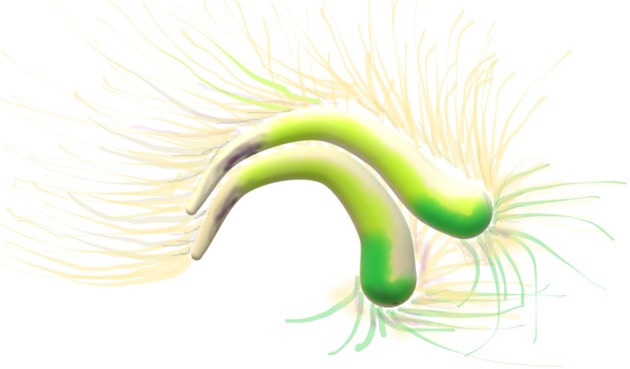
Diffusion tensor imaging, DTI image. Cingulum: two arches, the two C's yellow, green and beige. Thin axonal projections from the bilateral arches are visible.

✓ ***Functional connectivity***. Cerebral imaging techniques such as MRI can measure intrinsic hemodynamic signals linked to neural activity (functional MRI or fMRI). The structure of the matrix can reveal what information is encoded in a region by comparing it to other similarity matrices, such as from human judgments or computational models. As you will see, some authors are reporting real-time results, in neurofeedback studies, used by the participant to change their strategy or behavior.
✓ ***High-resolution anatomical mapping***. What makes diffusion MR imaging (dMRI) so unique is that during their diffusion-driven motions, water molecules are focusing on tissue structures at a micrometer scale far beyond the standard millimetric MR imaging resolution (LeBihan, 2013). The non-invasive observation of the water diffusion–driven motion distributions thus provides unique hints to the delicate structural features and geometric organization of tissues and the changes with physiologic or pathologic states (Figure 1, MD: mean diffusivity). The dMRI data also provides a direct view of the displacements of water molecules further reflecting the brain tissue microarchitecture (Le Bihan, 2003; Figure 2). In their study, Burzynska et al., used the fractional anisotropy (FA), a prevalent measure of the directional dependence of diffusion, reflecting fiber orientation, density to characterize white fibers tracts. Diffusion tensor imaging, DTI, is a 3D modeling magnetic resonance technique enabling white matter, WM, tractography and producing neural projections images, mapping axonal trajectories (Figure 2).
✓ ***Macro-networks***. Cerebral imaging provides a macroscopic cartography of evolving brain networks, related to the status creation-removal of synaptic connections in a macro-network (Foster, 2015). This is the first level of fractal scaling reflecting the number of projections to or from an area of the brain: regions of gray matter with high density of nodes and projections.
✓ ***Micro-networks***. Other fractal dimensions, at the sub-level of intra-cellular scale, via *micro-networks* or *interactome-networks* regulate the physiological mechanisms of the selection process in determining the preferential attachment of synapses and will affect by feedback the cartography of brain macro-networks (Foster, 2015).

### What Is Aging?

**Figure 3:**
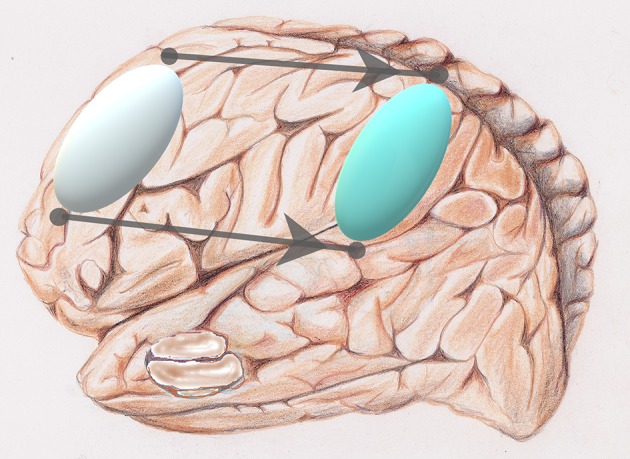
Reorganization: via a sort of “network translation” from frontal (silver) to parietal (light blue) of a privileged activation during aging.

✓ ***Hallmarks of cellular aging***. The time-dependent accumulation of cellular damage leading to a functional decline is considered to be the underlying starting point of aging. Hallmarks of the aging process such as genomic instability, telomere attrition, dysregulated energy metabolism, mitochondrial dysfunction, stem cell exhaustion shape the aging phenotype (Lopez-Otin et al., 2013; Mattson and Arumugam, 2018). Cellular senescence may be described as a durable arrest of the cell cycle coupled to stereotyped phenotypic changes and loss of functionality. In the brain, glial cell activation and inflammation are spreading (Mattson and Arumugam, 2018).
✓ ***Aging brain networks***. In another fractal dimension, on another scale, molecular networks of the human frontal cortex or the dorsal lateral prefrontal cortex, DLPFC, Figure 3 are directly associated with cognitive decline and other large-scale transcriptomic changes in the aging brain (Mostafavi et al., 2018). Decreased Intra-network functional connectivity of the salience, visual and sensorimotor networks decreased with aging (Lyoo and Yoon, 2017).

### Cognitive Decline Mitigated? Interventions

***Types of Interventions [Stimulation/Action*
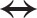
*Effects on Brain]***. This series of articles investigates the cognition performance in aging by neuropsychological tests related to cerebral imaging providing a status of the functional connectivity, brain networks and mapping. A first type of intervention is proposed, by stimulating the brain, *s*Brain (e.g., mental training, meditation and virtual reality/games), with direct effect on it, *e*Brain, noted as [***s*Brain 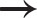
*e*Brain**]. The effect may be observed on the same function/task or same networks. Other [***s*Brain 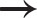
*e*Brain**] experiments seek to have a more general effect impacting functions different from the stimulation/training task and networks known to be involved in the processing. A second type of intervention, physical exercise, seems to be playing an instrumental role in the cognitive enhancement. It is still unclear how all underlying mechanisms of the activation of the cardiorespiratory and skeletal muscle systems, *a*CRM, have an effect on the brain, *e*Brain, designated as [***a*CRM 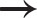
*e*Brain**]. However, mental training, meditation or virtual reality (films, games) require only minimal motor activity and cardio-respiratory stimulation. Therefore, other potential paths [***s*Brain 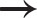
*e*Brain**] molding brain networks are nonetheless essential. Practical details on how to implement the various types of training programs evaluated by the authors such as mental, [***s*Brain 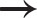
*e*Brain**] (memory, attention, mindfulness, neurofeedback, etc.) and cardiorespiratory-physical exercise, [***a*CRM 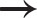
*e*Brain**] (aerobic, dancing, etc.) may be found elsewhere in books, websites, institutions, or training centers.

## Memory Training Programs

Requena et al. found in a randomized controlled trial that time-extended programs [6-year training] in healthy older people significantly improve everyday memory in contrast with the usual intensive programs whose effects decline with time. The time-extended program involves a group memory therapy based on the Wilson's model with cognitive and emotional content. A first series of modules encompasses strategies to activate the working memory, retrospective, and prospective. A second series of modules, discussion groups emphasize the mood in a social environment. Personal perspective confronted with that of others strengthens the training process.

Working memory (wm) is the process to temporarily store, maintain, and organize task-relevant information. The wm is an essential element in the preservation of age-related language understanding (Payne and Stine-Morrow). In their study (Payne and Stine-Morrow), examined the effects of a novel home-based computerized cognitive training program targeting verbal working memory in healthy older adults. Participants in the wm training group showed non-linear improvements in performance on trained verbal working memory tasks.

## Reorganization: Frontal to Parietal

Changes in synaptic connections are considered essential for learning and memory formation. However, it is unclear how neural circuits undergo continuous synaptic changes during learning while maintaining lifelong memories (Yang et al., 2009). Learning and novel sensory experience cause spine formation and pruning by a protracted process. Intensity of spine remodeling correlates with behavioral improvement after learning, underlying the essential role of synaptic structural plasticity in memory formation. The training-induced subset fraction of new spines along with long-lasting spines from the early development, surviving experience-dependent pruning, remains the structural basis for memory retention throughout the entire life. This is suggesting that lifelong memories are stored in stable connected synaptic networks (Yang et al., 2009).

Methqal et al. confirmed this observation, exploring the age-related, neuro-functional basis for executive semantic processing of words. Healthy aging is associated with neuro-functional reorganization that maintains cognitive performance. They employed a new word-matching task adapted for use in fMRI protocols. Such a task requires the flexible use of semantic relationship (or rules) supported by two executive processes with one invoking a higher-level of executive control. Results demonstrated that the shift in age-related brain activation from frontal to parietal regions (Figure 3) is a form of neuro-functional reorganization underlying executive processes during a word-matching task. Observing the maintain rule, which requires maintaining a given semantic rule through working memory updates, older adults demonstrated bilateral frontal activation, compared to more lateralized activations in younger adults. The posterior and dorsolateral prefrontal cortices were further recruited when older participants maintained and updated rule classifications in working memory. Disruption of one region results in shifting the recruitment in other parts of the network. Hence, a healthy aging brain recruits more than one pathway to preserve cognitive performance by recruiting the inferior parietal region when executive frontal resources are in high demand.

Jiang et al., developed a novel fMRI data analysis technique, local regional heterogeneity analysis, or *H*_*corr*_, to estimate neuronal selectivity based on fMRI activation patterns (Jiang et al., 2013). In their new study, Jiang et al., investigated whether this cutting-edge *H*_*corr*_ technique can effectively assess neuronal selectivity across brain regions in elders. The *H*_*corr*_-estimated neuronal selectivity may discriminate individual differences in behavioral performance on two cognitive functions, episodic memory, and letter verbal fluency (Jiang et al.). The authors assessed the neuronal selectivity for two brain regions (Jiang et al.), the hippocampus, which is associated with episodic memory and the visual word form area, VWFA, an area in the left ventral occipitotemporal cortex that is important for lexical aspects of language skills (Thielen et al.).

**Figure 4:**
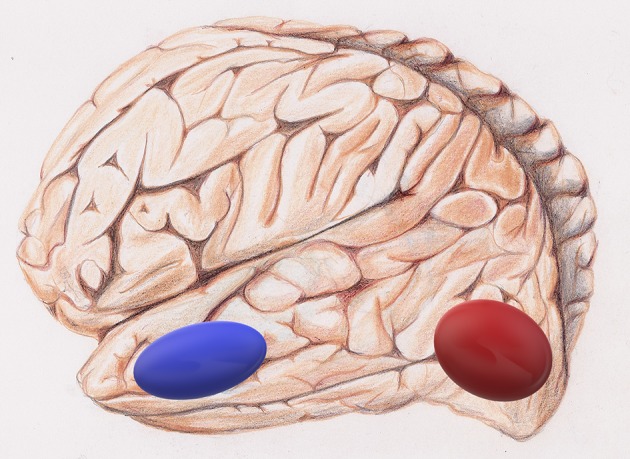
The visual word form area, VWFA, is in red and the hippocampus in blue.

They identified a double dissociation: episodic memory performance correlates with neuronal selectivity in the hippocampus, but not VWFA (Figure 4), whereas verbal fluency shows the reverse pattern. This suggests a direct relationship between cognitive function and neuronal selectivity at the corresponding brain regions in healthy older adults. The authors conclude that age-related brain networks differences might not compensate for cognitive decline in healthy older adults (Jiang et al.). Rather, they may contribute to this decline.

## Near Transfer

How “brain training” can improve cognition across several mental processes, as a general extension of its effects, measured as an improved performance on tasks differing from the trained tasks (transfer of training), is a debatable and hot topic (Souders et al.). Cognitive training, in the form of computer game training activities demonstrated some degree of success in the past and might result in broad transfer (Souders et al.). In their study, Souders et al., found that participants were learning specific skills and strategies from game training that later influenced steadily their performance on a similar task only. There was little evidence of transfer, even the near-transfer effect was weak (Souders et al.).

Shifting training in the elderly shows strong and long-lasting effects on the trained tasks albeit very little benefit in terms of generalization of ability to new tasks and activation of new brain networks (Gronholm-Nyman et al.). They made use of functions such as: (1) working memory updating, (2) inhibition of task-irrelevant responses, and (3) shifting between tasks and mental sets. In the computer setting that they used they found limited evidence for near transfer, and no far transfer based on an extensive test battery evaluating other executive domains such as fluid intelligence, episodic memory, verbal fluency, or visuomotor speed.

## Transcranial Direct Cerebral Stimulation, tDCS

Passow et al., assessed the effect of behavioral cognitive training and state-of-the-art of non-invasive brain stimulation techniques such as transcranial direct current stimulation, *tDCS*, in the condition of cognitive aging. They focused on working memory and episodic memory functions. The *tDCS*-induced subthreshold changes in neuronal resting membrane potentials alter the cortical excitability and activity, dependent on the direction of the current flow (Passow et al.). Studies of stimulating the human motor cortex have shown that anodal *tDCS* facilitates, while cathodal *tDCS* reduces excitability. The repetitive transcranial magnetic stimulations over prefrontal brain regions cause modulatory effect on dopaminergic neurotransmission. Clearly, a “central executive brain network” is responsible for supervising the integration of information and for coordinating “slave systems” that are responsible for the short-term maintenance of information. The central executive is responsible inter alia for directing attention to relevant information, suppressing irrelevant information and inappropriate actions, and coordinating cognitive processes when more than one task is simultaneously performed.

## Attention, as an Essential Mechanism

Several studies published in this book investigate the role of attention in learning and preserving cognitive abilities. Attention is the process of selectively concentrating on a specific aspect of information, while disregarding the noise from other perceivable information. Specifically, Ayasse et al., found that eye fixations on a referenced object in a spoken sentence occurred as rapidly in elders than younger adults. However, in elders, the executive processing resources were slower in indicating the referenced object with a precise response (Ayasse et al.). Furthermore, the hearing process did not seem to play a limitation as would be expected in hearing-impaired elders.

### Visual Spatial and Temporal Attentions

In contrast, visual search performance is thought to decline with age when the target is visually indistinct from distractors and a serial search is required (Callaghan et al.).

It is well-established that older adults require longer to process visual stimuli—i.e., have slower processing speeds and display an increased magnitude of the so-called “attentional blink” (Bateman et al., 2009; Callaghan et al.). Visual spatial attention aims the scrutiny to a particular location in space, whereas visual temporal attention focuses the attention to specific instants of time. The authors found larger switch-costs between temporal and spatial attention in older adults compared to younger (Callaghan et al.).

## Attention, Aging, and Brain Network Reorganization

**Figure 5:**
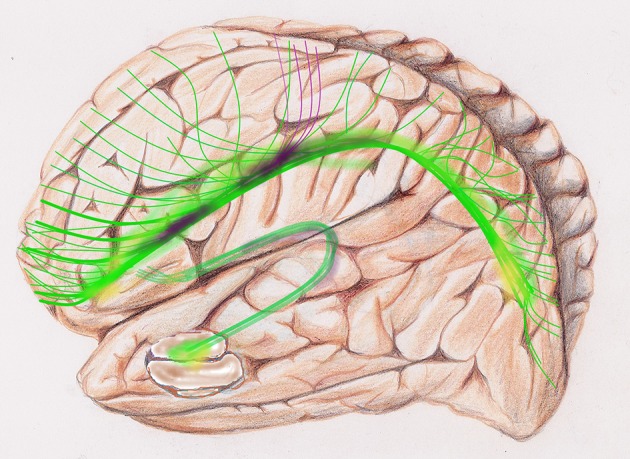
Global tractography, sketch of diffusion tensor images, DTI, of the default mode network, DMN: is a set of areas active at rest. The upper C-shaped arch (green-yellow-purple) with widespread projection bundles (green-purple) is the cingulate cortex. The cingulum bundle radiates far-reaching antero-posterior connectivity from medial prefrontal lobe to the precuneus and other areas of the brain, e.g., frontal, parietal. The fornix, a lower and smaller C-Shaped arch is a major output projection tract of the hippocampus.

The degree of connectivity between the frontoparietal control network, FPN, and the default mode network, DMN, decreased with age from younger to older adults (Figure 5). The connectivity between FPN and dorsal attention network, DAN, remains stable across age groups (Avelar-Pereira et al.). This suggests that dynamic interactions of the FPN are stable across cognitive states. The DMN and DAN were anti-correlated with a task-dependent age-sensitivity. This anticorrelation increased from rest to task. Interestingly, the degree of DMN-DAN anticorrelation was associated to resting cerebral blood flow within the DMN. This implies that reduced DMN neural activity during rest underlies an impaired ability to achieve high levels of anticorrelation during a task (Avelar-Pereira et al.). Furthermore, there is a switching role for the FPN by dynamically interacting with DMN and DAN depending on task demands. Nodes changed their network affiliation and showed realignment from rest to task, implying a more flexible connectivity profile (Avelar-Pereira et al.). The authors found that older adults had lower FPN-DMN functional connectivity during both rest and the MSIT, but yet expressed greater FPN-DMN connectivity at rest compared to task (Avelar-Pereira et al.). The FPN serves as a switch to actively engage other networks and facilitate cognition in older adults. Normal aging is accompanied by a lower degree of flexible network interactivity (Avelar-Pereira et al.).

The overarching question is whether and how neurovascular coupling is produced during learning (Mitra and Raichle, 2016), i.e., correlating the brain network activation to the cerebral blood flow, CBF. Evidence for propagating low frequency activity underlies the neurovascular coupling and implies that the BOLD signal propagation is likely of neural origin (Mitra and Raichle, 2016). Vigilance creates an asymmetry with increase of left hemisphere activation in young adults. Aged-induced remodeling of brain networks underlying vigilance, the “hemispheric asymmetry reduction in older adults,” or HAROLD model is supported by functional neuroimaging (Cabeza, 2002; Cabeza et al., 2002). In their study, Harwood et al., investigated whether the HAROLD brain networks age-induced reorganization, i.e., asymmetric reduction, or increase of bilateralization, result from a compensatory effect to attain a similar level of vigilance and cognitive performance compared to young adults (Harwood et al.). Making use of functional transcranial Doppler, fTCD, Harwood et al. found that CBF velocity, CBFV, declined during aging, and only young adults showed the typical right-lateralized CBFV pattern. Older adults showed greater left hemisphere activation consistent with the HAROLD model. However, the increased left hemisphere activation did not appear to be compensatory as the older adults performed at a significantly lower vigilance level compared to young adults.

## Neurofeedback

*Neurofeedback (NFB)* monitors real-time displays of brain activity, e.g., electroencephalography (EEG), to enhance brain function and behavioral performance on a positive self-regulating feedback mode. Characteristic brainwave measurement is training technique to enhance performance in athletes and musicians, creativity, attention and working memory (Daly and Wolpaw, 2008; Jiang et al.; Reis et al.). Typically, EEG theta oscillations are related to hippocampal activity during working memory (Tesche and Karhu, 2000). Spatial attention is a constant theta-rhythmic sampling process implemented through gamma-band synchrony (Landau et al., 2015). Two striking studies in this book might disrupt the classic thoughts about near-transfer spreading and generalization of training to other brain networks and tasks (Jiang et al.; Reis et al.).

Reis et al., found cognitive performance improvements after 8 consecutive days of training in specific tasks. NFB training led to the ability to uniquely up-modulate both alpha and theta frequency bands and seemed to produce positive effects in cognitive performance. The analysis of the EEG acquired before and after NFB training, during the computerized battery, revealed that only the participants under NFB training were able to increase alpha and theta rhythms from pre- to post-training. Remarkably, Reis et al., identified a positive correlation between a successful (theta) NFB training and a better performance in specific tasks, as well as an increased alpha activity between pre- and post-training. Considering the overall functional system brain networks, it entails that EEG-NFB training might be *generalized and not restricted to the region of training or limited to a near-transfer* (Reis et al.).

In their article “Tuning Up the Old Brain with New Tricks,” Jiang et al., are assessing how NFB training of older brains may be aiming to match those of younger brains during attention/working memory tasks. Jiang et al., conclude that NFB associated with new neurological measurements, e.g., “neuromarkers” such as event-related potentials and connectivity, is providing new hope for brain and cognitive training in the growing older population. This is a promising roadmap for future work.

## Mindfulness and Transcendental Meditation

**Figure 6:**
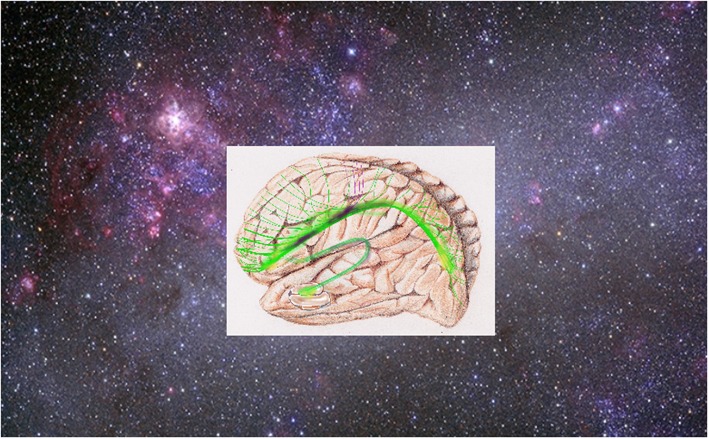
Transcendental meditation: “it is sometimes qualified as a cosmic state of mind, harmonious with the rhythm of nature and cosmic life. The transcendental meditation brings the individual into a wider cosmic dimension and consciousness” (Yogi, 1973). Photo: Credit NASA, Goddard Space Flight Center and Penn State University.

Mindfulness is the psychological process of capturing a person's attention to the present moment. Practice of meditation trains to focusing on the present instant clearing the mind form past experiences and future possibilities (Figure 6). Mindfulness is derived from “*sati*,” a *Sanskrit* word, meaning “awareness,” based on “*Zen*” in the Buddhist tradition. Spiritual leaders such as the Dalai Lama and Thích Nhãt Hạnh gave rise to the popularity of mindfulness in the modern western culture. Mindfulness, as a modus operandi to mitigate cognitive impairment is described in two articles of this book.

Mindfulness training positively influences three essential domains of aging: (1) behavioral and neural correlating with attentional performance; (2) psychological well-being; and (3) systemic inflammation (Fountain-Zaragoza and Prakash). The scientific focus on mindfulness training is still recent, with limited randomized controlled trials utilizing active comparison conditions. Based on preliminary results, transcendental meditation seems to be more efficient than mindfulness, but both were worthier than relaxation or absence of treatment. Mindfulness and transcendental meditation associated with cognitive training, similarly improved word fluency. This preliminary evidence for cognitive benefits following transcendental meditation, has direct parallels with the current mindfulness training approaches (Fountain-Zaragoza and Prakash). Preliminary cross-sectional evidence suggests that mindfulness is associated with enhanced psychological well-being, measured as self-reported depressive symptoms, quality of life, and stress, in the elderly (Fiocco and Mallya, 2015) and emotion regulation mediates the relationship between mindfulness and reduced perceived stress across age (Prakash et al., 2015). Emotional distress and/or psychopathology can be accompanied by changes in inflammatory processes, which are further entailing several health issues. Participating in a mindfulness-based stress reduction program led to significant down-regulation of the pro-inflammatory gene *NF-*κ*B* in leukocytes and the NF-κB (Creswell et al., 2012; Fountain-Zaragoza and Prakash).

Active experiencing (AE) is a form of mindfulness training improving attentional control to mitigate cognitive decline with via an immersive acting program. Unlike mindfulness meditation, participants in AE have a specific task to engage and follow instructions during every rehearsal. Episodic memory is particularly vulnerable to decline in aging (Banducci et al.). In their study, Banducci et al. identified specific gains in episodic recall from AE compared to active individuals (“control” group), albeit they found no other evidence of AE-intervention gains in cognition. AE produced greater gains persistent up to 4 months after AE-intervention. Intervention conditions were similar in magnitude across gains in working memory, executive function and processing speed (Banducci et al.).

## Foreign-language

Because most cognition processing, or at least some of them, appear to eventually be accessible to sharing with others, via word-description (Hauser et al., 2002), the paramount question is where is the neuroanatomical location recruited by the language? It entails the hypothesis that the activation in a new language of areas of the brain in a broader sense may enhance the brain training [_**s**_**Brain 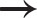
**_**e**_**Brain**], leading to better results of cognition preservation in older adults. Foreign-language acquisition has been shown to demonstrate structural neuroplasticity in children, adults, and elderly, even after short-term language training (Hosoda et al., 2013; Pliatsikas et al., 2015, 2017). In a community-dwelling, *not immersion* in a foreign country, learning a foreign language later in life could thus strengthen cognitive functioning in older adults as Ware et al., investigated in their study. The cognitive and loneliness perception scores did significantly improve in their study albeit language learning became a benefit as a social activity. Participants noted that differences in foreign language fluency encouraged seeking for help and fostered social interaction (Ware et al.). These results are not surprising. According to Noam Chomsky past the very earliest developmental stages, much of further acquisitions will be gained by imitation (Chomsky, 1964). Therefore, it takes more than practice for a second language to share native-like features such as easiness, fluency.

## Physical Activity and Communication Brain-Body: Unpicking the Riddle of Cause and Effect

Two postulates about [***a*CRM 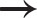
*e*Brain**] and [***s*Brain 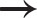
*e*Brain**] may be formulated. *A first postulate*: strikingly, the activations of the cardiorespiratory and skeletal muscle systems, *a*CRM, seem to produce *a generalized effect on the brain, e*Brain, [***a*CRM 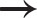
*e*Brain**], *across broad aspects of cognitive performance* (Colcombe et al., 2004, 2006; Foster et al., 2011; Foster, 2015; Jonasson et al.). Would an exercise-induced overall downregulation of brain amyloid β (Aβ) levels by increased clearance via the choroid plexus (Carro et al., 2002; Adlard et al., 2005) be producing this general effect? A deficient clearance of Aβ_1−42_and Aβ_1−40_ in the brain via the choroid plexus also contributes to Alzheimer's disease-related amyloidosis deposition (Carro et al., 2002, 2006; Mawuenyega et al., 2010; Foster et al., 2011). *A second postulate*: In contrast, task-induced direct stimulation of the brain, *s*Brain (e.g., mental training, meditation, and virtual reality/games), **[*s*Brain 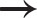
*e*Brain]**, seems to produce localized effects to the brain, *e*Brain, *restricted to the networks associated to the task-training* (Anguera et al., 2013; Foster, 2015; Gronholm-Nyman et al.; Souders et al.). Indeed, the **[*s*Brain 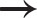
*e*Brain]** modality appears to produce localized effects with little or absence of near-transfer, and no broad-transfer, i.e., absence of gain on tasks differing from training and on non-activated brain networks. Further studies to the ***[sBrain*
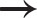
*eBrain]-postulate***are necessary to investigate how to cross these boundaries.

Supporting the first postulate a study demonstrated that aerobic exercise has the potential to broadly improve cognition and reduce brain atrophy in older adults (Jonasson et al.). They concluded that aerobic exercise in sedentary older adults has the potential to comprehensively improve cognition, rather than circumscribed to a specific skill/brain network, as captured by a “cognitive score” across cognitive capabilities based on episodic memory, processing speed, working memory updating, and executive function tasks. A one-time intervention, limited span of 6-month aerobic training, seemed insufficient to produce a notable direct effect on the prefrontal cortical thickness (Jonasson et al.). Rather, aerobic fitness at baseline was specifically related to larger thickness in dorsolateral prefrontal cortex (DLPFC), and hippocampus volume. The increased thickness appeared to be positively associated with increased aerobic fitness over a long time span (>12 months). It may be hypothesized that exercise-induced increased cortical thickness or reduced cortical thinning phenomenon localized to DLPFC (Jonasson et al.) and its potential further extension to other regions of the cortex is a slow process, undetectable within 6-months, requiring an extended period of time.

However, aerobic performance (VO_2max_) may not be the limiting factor; other factors closely related to aerobic performance might be involved, e.g., cardiorespiratory health status, [***a*CRM 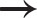
*e*Brain**], or else [***s*Brain 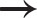
*e*Brain**]. The level of physical exercise inherent to dancing may be insufficient to produce an increase in aerobic fitness (VO_2max_) albeit the exercise-induced elevation of heart rate, cardiac output. The perfusion may have been sufficient to produce changes in protein expression (brain-derived neurotrophic factor, BDNF; cytokines; insulin-like-growth factors, IGF-1 and IGF-2) improving cognitive plasticity (Chen et al., 2011; Foster et al., 2011; Foster, 2013). Dancing involves the moving, spinning, swaying, swinging, twisting, rocking, in a three-dimensional space which underlies brain activation of the path-integration maintaining permanent visual tracking of the direction and distance from reference points (landmark) during 3-D navigation in the environment and thus requires the activation of hippocampal and entorhinal networks (Foster, 2013). In dancing, information from the body position, movement (e.g., limbs, head, and trunk), and accelerations are integrated in the CNS along with instructions from the vestibular system and cerebellum, all of which contributing to create a spatial representation in real-time. In this case, the activation cascade becomes [***a*CRM 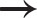
*s*Brain 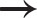
*e*Brain**], *a*CRM being only a modus vivendi, instrumental at automatically operating the indirect *s*Brain activation causing the expected cognitive effects, *e*Brain (Foster, 2013, 2015). Therefore, the absolute (VO_2max_) value may bear a variable-spectrum of influence, from high to absence in preserving or promoting cognitive performance. Clearly, depending on several cofactors, the activations of the cardiorespiratory, skeletal muscle systems, and movements trigger other cognitive performance promoters.

In the study by Burzynska et al., (VO_2max_) played little or no influence. They focused on the white matter (WM) microstructure integrity, since the degeneration or loss of axons and myelin is considered as a central mechanism underlying age-related cognitive decline (Gunning-Dixon and Raz, 2000). Extensive and concurrent decreases in FA, and modification of other dMRI factors are associated to WM degeneration, loss of myelin or axonal integrity. Burzynska et al., observed a decline in WM integrity across the majority of brain regions unless 6-month dance-intervention was performed. A one-time intervention, limited span of 6-month dancing seemed insufficient to produce an effect on post-intervention processing speed. Walking alone, walking associated with nutrition, or being an active elder produced no effect and were insufficient to prevent the decline of WM, including in the fornix. Therefore, preventing age-induced “WM structural disconnection” or improving WM integrity is key in preserving cognitive performance necessary for independent functioning in the elderly (Burzynska et al.).

## Obesity: How Much Can We Learn About the Brain?

We may deduce remarkable insights on the body–brain connections by analyzing the physiopathology of obesity. The thorough examination by Stillman et al., in their review “effects of obesity and behavioral interventions on neurocognitive aging” (Stillman et al.) provides clues to the [***a*CRM 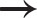
*e*Brain**], [***s*Brain 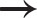
*e*Brain**], [***a*CRM 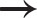
*s*Brain 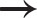
*e*Brain**], and **diet** postulates.

### Gray Matter Reduction

Several studies demonstrated that physical activity [***a*CRM 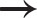
*e*Brain**], is associated with increased gray matter (GM) volume in the hippocampus and prefrontal cortex (Colcombe et al., 2004; Erickson et al., 2011). The obesity-induced reduction of GM volume is found in several brain regions, including the hippocampus, prefrontal cortex, and other subcortical regions (Stillman et al.). They identified a characteristic role of excess body fat in the atrophy of GM, independently of other variables such as other comorbid conditions, e.g., diabetes.

### White Matter in Obesity: Quantity vs. Quality?

Indeed, the structural integrity of the WM tracts in the connectome is reduced in obesity, recent DTI work has shown an obesity-induced decrease in FA. This pattern is associated with demyelination in models of inflammatory disorders, underlying a systemic increase in circulating inflammatory cytokines reported in obesity. Presence of white matter hyperintensities (WMH) might be indication of demyelination processes (Stillman et al.).

In summary, obesity is associated with an altered integrity of GM and WM in a process similar to that observed in normal aging and obesity is likely to accelerate brain aging (Stillman et al.).

### Functional Brain Activation

Stillman et al., analyze how obese adults consistently exhibit hyperactivation to food compared to non-food elements in the limbic-orbitofrontal areas (fMRI) associated with decision making, satiety, motivation, and reward compared to healthy-weight controls. This hyperactivation interferes and potentially disrupts brain networks involved in executive functioning and memory. Physical activity may not be a necessary component to interventions designed to reverse obesity-related neurocognitive dysfunction. Cognitive performance seems related to cardiovascular fitness rather than to weight and adiposity. Clearly, weight loss may not be a requirement to mitigate the negative neurocognitive consequences of obesity.

Physical activity [***a*CRM 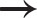
*e*Brain**], has consistent effects on the hippocampus and prefrontal cortex, as well as the white matter tracts connecting these regions (Stillman et al.). Physiologically, physical activity operates in the reverse direction to obesity.

Dietary interventions do not increase cardiovascular fitness, yet improve cognitive performance (Stillman et al.). Dietary restriction such as intermittent fasting into the lifestyle improves cognitive capabilities and neuronal resilience (Mattson, 2019).

The “obesity paradox,” valid for the elderly group [>73 yrs.], referred to as the “oldest old,” the classic scheme does not strictly apply. Higher BMI appears to bear protective effects on the cognitive performance in “oldest old” (Hainer and Aldhoon-Hainerova, 2013; Stillman et al.).

## FDA Clearance for Medical Software/Device

Approximately 20 companies designing computerized cognitive training (CCT) will conduct clinical trials to prove their efficacy and obtain the green light from Food and Drug Administration (FDA) clearance as a medical software/device (Motter et al.). In their survey, Motter et al. are describing the state-of-the-art cognitive training, the clinical trials, and the future trends.

## How and Why Does Our Internal Perception of Time Vary With Age?

Who has not heard elders say, “*time has just been flying by*,” “*time seems to whiz by faster and faster as we get older?*” In a paper the authors (Turgeon et al.), highlight that a hallmark of normal aging is the increased noise and temporal uncertainty as a result of impairments in attention and memory. The authors present the potential reduction in accuracy of the central timing by underlying mechanisms of dopamine-glutamate interactions in cortico-striatal circuits. In relation to these observations, the authors propose potential interventions that may reduce the prospect of age-related declines in timing consciousness.

## Author Contributions

All authors listed have made a substantial, direct and intellectual contribution to the work, and approved it for publication.

### Conflict of Interest

The authors declare that the research was conducted in the absence of any commercial or financial relationships that could be construed as a potential conflict of interest.
